# Humans Strengthen Bottom-Up Effects and Weaken Trophic Cascades in a Terrestrial Food Web

**DOI:** 10.1371/journal.pone.0064311

**Published:** 2013-05-08

**Authors:** Tyler B. Muhly, Mark Hebblewhite, Dale Paton, Justin A. Pitt, Mark S. Boyce, Marco Musiani

**Affiliations:** 1 Faculty of Environmental Design, University of Calgary, Calgary, Alberta, Canada; 2 Ecosystem Management, Alberta Innovates – Technology Futures, Vegreville, Alberta, Canada; 3 Department of Ecosystem and Conservation Sciences, University of Montana, Missoula, Montana, United States of America; 4 Department of Biological Sciences, University of Alberta, Edmonton, Alberta, Canada; Umea University, Sweden

## Abstract

Ongoing debate about whether food webs are primarily regulated by predators or by primary plant productivity, cast as top-down and bottom-up effects, respectively, may becoming superfluous. Given that most of the world's ecosystems are human dominated we broadened this dichotomy by considering human effects in a terrestrial food-web. We studied a multiple human-use landscape in southwest Alberta, Canada, as opposed to protected areas where previous terrestrial food-web studies have been conducted. We used structural equation models (SEMs) to assess the strength and direction of relationships between the density and distribution of: (1) humans, measured using a density index; (2) wolves (*Canis lupus*), elk (*Cervus elpahus*) and domestic cattle (*Bos taurus*), measured using resource selection functions, and; (3) forage quality, quantity and utilization (measured at vegetation sampling plots). Relationships were evaluated by taking advantage of temporal and spatial variation in human density, including day versus night, and two landscapes with the highest and lowest human density in the study area. Here we show that forage-mediated effects of humans had primacy over predator-mediated effects in the food web. In our parsimonious SEM, occurrence of humans was most correlated with occurrence of forage (β = 0.637, *p*<0.0001). Elk and cattle distribution were correlated with forage (elk day: β = 0.400, *p*<0.0001; elk night: β = 0.369, *p*<0.0001; cattle day: β = 0.403, *p*<0.0001; cattle, night: β = 0.436, *p*<0.0001), and the distribution of elk or cattle and wolves were positively correlated during daytime (elk: β = 0.293, *p* <0.0001, cattle: β = 0.303, *p*<0.0001) and nighttime (elk: β = 0.460, *p*<0.0001, cattle: β = 0.482, *p*<0.0001). Our results contrast with research conducted in protected areas that suggested human effects in the food web are primarily predator-mediated. Instead, human influence on vegetation may strengthen bottom-up predominance and weaken top-down trophic cascades in ecosystems. We suggest that human influences on ecosystems may usurp top-down and bottom-up effects.

## Introduction

Food-webs may be influenced by both bottom-up effects that link plants to herbivores and higher trophic levels and by top-down effects from carnivores to plants. However, there is still significant debate about the relative importance of each in ecosystems. Recent studies on large mammal food webs in protected areas indicate that predators can have strong top-down effects on prey and indirectly on vegetation [1], [2], i.e., “trophic cascades” [3]. This has led some ecologists to argue that top-down effects of top carnivores have primacy in driving food-web dynamics [4], and rising popularity of top-down forces in the popular literature [5]. In reality, top-down and bottom-up mechanisms operate simultaneously in food webs [6]. Emphasizing the importance of one mechanism over the other may falsely dichotomize how ecosystems function.

Importantly, oversimplifying ecosystem dynamics into top-down effects of predators versus bottom-up effects of plant biomass availability could be underestimating the importance of human influences in ecosystems. Human influences on food webs likely are pervasive and may operate at multiple trophic levels simultaneously. For example, humans have modified ecosystems for tens of thousands of years through bottom-up (i.e., ecosystem engineering [7]) and top-down effects (i.e., overharvest and continental-scale megafaunal extinctions [8], [9]). Human impacts on ecosystems have intensified with a growing human population and demand for resources [10]. Thus, a contemporary challenge is to understand how growing human influences on ecosystems might propagate through food webs.

Here we describe and quantify human effects at multiple trophic levels of a terrestrial food web in a multiple human-use area, as opposed to protected areas where many previous terrestrial food web studies have been conducted. We show that human effects were pervasive on the studied species in the food web, and manifested through both direct and indirect pathways as they propagated across trophic levels. We show that indirect effects of humans were forage-mediated rather than predator-mediated. We conclude that pervasive effects of humans in ecosystems can perturb top-down and bottom-up effects in food-web dynamics.

## Methods

The study occurred in a 9,000 km^2^ area of southwest Alberta, Canada [11]. We used structural equation models (SEMs [12]) to assess the strength and direction of the spatial relationships between the occurrence of: (1) humans, measured using a density index; (2) wolves (*Canis lupus*), elk (*Cervus elaphus*) and cattle (*Bos taurus*), measured using telemetry data and resource selection functions (RSFs), and; (3) forage quality and quantity and utilization (measured at vegetation sampling plots). In selecting study species, we followed previous studies, which used human-wolf-ungulate-vegetation dynamics to illustrate the importance of top-down versus bottom-up effects in terrestrial ecosystems [1], [2]. In our study, wolves were the predominant predator of the two dominant herbivores, one native, elk, and one non-native, cattle. Relationships between species occurrence were evaluated by taking advantage of pseudo-experimental temporal and spatial variation in human activity, including: (a) day versus night, and (b) in two landscapes with the highest and lowest human density in the study area. Testing these relationships across different levels of human activity allowed us to assess the sensitivity of species co-occurrence to human activity. If human effects on the food web were forage-mediated, we predicted a positive influence of humans on forage, of forage on herbivores (i.e., elk and domestic cattle), and of herbivores on wolves. Alternatively, if human effects were predator-mediated, we predicted a negative influence of humans on wolves, wolves on herbivores and herbivores on forage.

We produced a spatial index of human density in a Geographic Information System (GIS), validated with actual counts of humans obtained using trail cameras (RECONYX Silent Image^TM^ Model RM30, *n* = 43) and pneumatic road tube traffic counters (Diamond Traffic Products, *n* = 43) deployed on spatially randomized roads and trails across the study area in 2008. We calculated the human density index based on travel time required to access any point in the study area along existing road and trails from human population centers (>100 people), given typical travel speeds and a decay exponent of –1.45 based on typical human recreational behavior [13]. We tested whether the human density index predicted actual counts using linear regression.

We obtained satellite and Global Positioning System (GPS) telemetry location data from wolves, elk and cattle and developed population-averaged RSFs [14], [15] to estimate habitat selection and the resultant predicted spatial distribution of each species. We obtained 7,462 locations from 14 wolves fitted with ARGOS satellite telemetry collars between 2004 and 2007, 267,440 locations from 62 elk fitted with GPS or ARGOS satellite telemetry collars (Lotek 4400 and 2200 series and Telonics TGW-3600) between 2007 and 2009, and 348,514 cattle location from 50 cattle GPS telemetry collars (Lotek 3300L) deployed within two separate grazing allotments between 2004 and 2007. Animal capture protocols were approved by the Universities of Alberta and Calgary and the government of Alberta (Permit Numbers: BI-2008-19, RC-06SW-001 and 23181CN).

RSFs were estimated using logistic regression in STATA 10.1 [16], where resources at telemetry locations are compared to resources at “available” locations [14], [17]. Available resource locations were sampled at random in each individual animal home range for wolves and elk, estimated using a 95% kernel density estimator of location data [18], or fenced pasture for cattle. We followed the “two-stage” method to calculate population-level RSFs [15], [19] where an RSF is calculated for each individual animal and these are averaged across all individuals. We produced daytime and nighttime RSFs because human counts were statistically different between night and day, and we hypothesized that wolves [20] and elk [21], might respond to these temporal changes. We validated RSF models using k-fold cross validation [22], [23].

We calculated a spatial index of forage quality and quantity from remotely sensed vegetation data. We obtained a 30-m^2^ spatial resolution GIS map of vegetation cover derived from Landsat data [24] and collapsed it into two ungulate food quality classes, high and low [11]. We multiplied this by the normalized difference vegetation index (NDVI) value at each pixel as an index of forage biomass [25]. This provided an index of forage quality (type) and quantity (productivity) at each pixel on the landscape.

We measured forage utilization by herbivores in 2007 and 2008 at 150 plots using the ocular estimate-by-plot method [26]. At 40 of the 150 plots, forage utilization was also assessed using the paired-subplot method [27] in which vegetation biomass on an area exposed to herbivory was compared to a nearby area where grazing was excluded with a 1.2 m×1.2 m cage. We assessed the accuracy of our visual estimates of utilization by comparing visual estimates to actual clipped biomass utilization values in a linear regression.

We overlaid our spatial models in a GIS and at each 30-m pixel along roads and trails (*n* = 760,140) we sampled the human index, RSF values and forage index. At each forage utilization plot we sampled RSF values for each species. SEMs were fit to these data using LISREL 8.72 [28]. Humans were modeled in SEMs as an exogenous variable, i.e., starting points of the model, whereas other species were endogenous variables, i.e., determined by pathways in the model, and that can also serve as predictors for other endogenous variables. SEMs were used in an exploratory mode where initial theoretical models of bottom-up and top-down only pathways were altered to improve the model fit by removing pathways for which the association was non-significant [29]. We assessed SEM fit using the goodness-of-fit index (GFI), adjusted goodness-of-fit index (AGFI), and root mean squared residual (RMR). A GFI>0.9, AGFI>0.9 and with a similar value to the GFI and RMR<0.05 indicate the model had a good fit to the data [30]. The best model had the lowest Akaike Information Criterion (AIC), i.e., parsimoniously fit the data, had significant interaction paths and fit the data.

## Results

**Figure 1:**
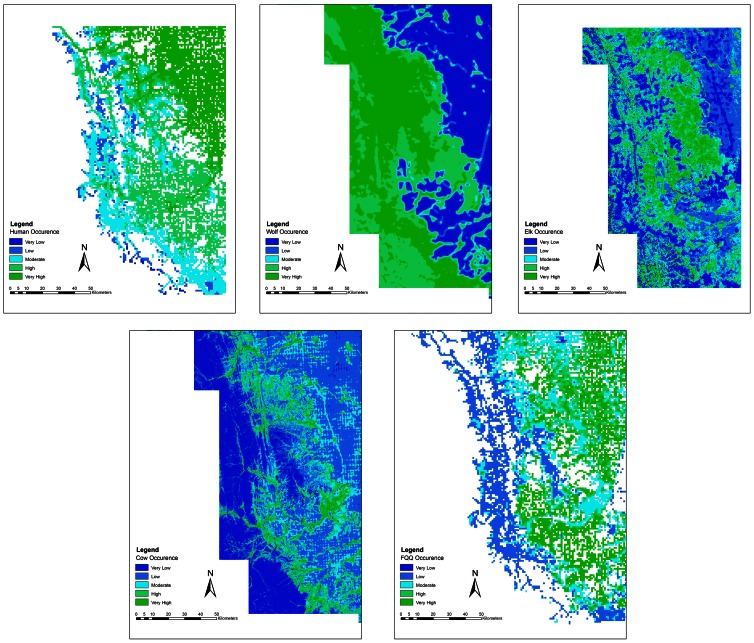
Spatial models of human (top left), wolf (top middle), elk (top right), cattle (bottom left) and forage quality and quantity (FQQ, bottom right) occurrence during the day in southwest Alberta, Canada. Low values (dark blue to light blue) indicate very low to moderate probability of occurrence at a location whereas high values (light blue to dark green) indicate moderate to very high probability of occurrence.

**Figure 2:**
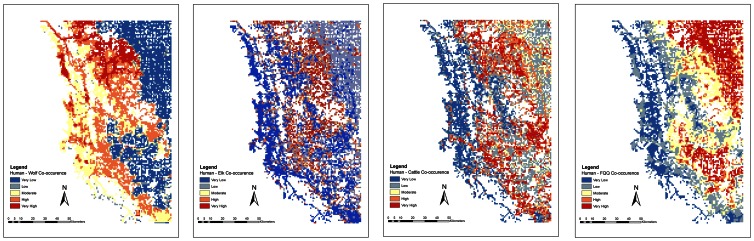
Co-occurrence of humans and wolves (left), humans and elk (middle left), humans and cattle (middle right) and humans and forage quality and quantity (FQQ, right), during the day along roads and trails in southwest Alberta, Canada. Low values (blue to yellow) indicate that the distribution is different (i.e., avoidance) at a location whereas high values (yellow to red) indicate that the distribution is similar (i.e., co-occurrence). For illustrative purposes roads and trails are exaggerated with a 1-km buffer.

Species density and distribution models were predictive of actual species occurrence. We confirmed a significant linear relationship between the human density index and actual number of humans counted on roads and trails at counter locations during the day (*R^2^* = 0.61, *F* = 197.78, *p*<0.001; Fig. 1) and night (*R^2^* = 0.57, *F* = 169.85, *p*<0.001). Population-level RSFs for wolves, elk and cattle (Table 1) were highly predictive of each species' distribution (Table 2). We found a significant linear fit (*R^2^* = 0.255, *F* = 47.50, *p*<0.001) between visual estimates of forage utilization to actual clipped biomass utilization values. We illustrated species co-occurrence with humans across the landscape by re-scaling each occurrence model index from 0 (low probability of occurrence) to 1 (high probability of occurrence) and adding species occurrence models together (Fig. 2).

**Table 1:** Coefficients for population-level resource selection function (RSF) models of wolves, elk and cattle during day and night in southwest Alberta, Canada.

	Wolf							Elk					Cattle				
	Day				Night			Day		Night			Day			Night	
Covariate	β			SE	β	SE		β	SE	β	SE		β		SE	β	SE
Road and Trail Density (km/km^2^)	0.118			0.1946	0.384	0.1907		–0.095	0.0700	–0.107	0.0773		0.290		0.1186	0.296	0.1236
Road and Trail Density^2^	–0.119			0.0478	–0.165	0.0551		–0.031	0.0144	–0.036	0.0152		–0.023		0.0232	–0.030	0.0273
Slope (°)	0.286			0.0716	0.063	0.0468		0.126	0.0099	0.122	0.0098		–0.070		0.0188	0.091	0.0327
Slope^2^	–0.010			0.0022	–0.007	0.0022		–0.004	0.0003	–0.004	0.0002		–0.002		0.0009	–0.008	0.0016
Distance to Water (m)	0.066			0.0220	0.038	0.0168		–0.000	0.0000	–0.000	0.0000		–0.000		0.0001	–0.000	0.0001
Treed Wetland	Dropped				–0.001	0.0002		Dropped		Dropped			Dropped			Dropped	
Open Wetland	Dropped				Dropped			Dropped		Dropped			Dropped			Dropped	
Dense Conifer Forest	0.054		0.0215		0.036	0.0166		0.119	0.5207	0.810		0.5432	–0.836	0.2694		–0.793	0.2982
Moderately Closed Canopy Conifer Forest	0.055		0.0212		0.037	0.0164		0.567	0.5171	1.151		0.5836	–0.070	0.2330		–0.302	0.2622
Open Conifer Forest	0.059		0.0219		0.040	0.0169		1.524	0.5155	2.291		0.5878	0.572	0.2288		1.289	0.2826
Mixed Forest	0.055		0.0213		0.037	0.0165		0.739	0.5068	1.439		0.5776	0.458	0.2217		0.510	0.2462
Broadleaf Forest	0.058		0.0213		0.035	0.0164		1.320	0.5218	2.111		0.5659	1.092	0.2795		0.623	0.2630
Regen	0.071		0.0206		0.050	0.0133		0.118	0.0560	–0.384		0.0743	Dropped			Dropped	
Shrub	0.054		0.0214		0.037	0.0165		1.026	0.5268	1.696		0.5871	0.855	0.2167		1.258	0.2401
Herbaceous	0.054		0.0211		0.036	0.0164		1.332	0.5110	2.043		0.5867	0.901	0.2447		1.470	0.2426
Agricultural Field	–0.015		0.0041		–0.014	0.0082		0.554	0.0831	0.666		0.1127	Dropped			Dropped	
Barren Ground	0.050		0.0207		0.035	0.0149		0.257	0.3540	1.238		0.4835	–2.313		0.5872	–1.750	0.4711
Snow/Ice	Dropped				Dropped			–0.677	0.3451	1.621		0.4561	Dropped			Dropped	
Intercept	–188.920	72.7008			–125.986		56.0790	–2.569	0.5722	–4.185		0.6555	–0.236		0.7997	–1.913	0.7578

**Table 2:** Results from 5-fold and pasture cross validation of wolf, elk and cattle resource selection functions (RSFs) during day and night in southwest Alberta, Canada.

	Wolf		Elk			Cattle	
	Day	Night	Day	Night		Day	Night
Spearman *r_s_*							
Group					Pasture		
1	1.000	1.000	1.000	1.000	Bob Creek – Porcupine Hills	0.726	0.808
2	0.988	0.976	1.000	1.000	Porcupine Hills – Bob Creek	0.618	0.599
3	0.964	0.988	1.000	1.000			
4	0.952	0.988	1.000	1.000			
5	0.994	0.951	1.000	1.000			
Mean	0.979	0.981	1.000	1.000		0.672	0.704
*R^2^*							
1	0.941	0.914	0.948	0.925	Bob Creek – Porcupine Hills	0.559	0.519
2	0.863	0.794	0.945	0.911	Porcupine Hills – Bob Creek	0.122	0.025
3	0.769	0.761	0.945	0.938			
4	0.874	0.841	0.949	0.926			
5	0.857	0.839	0.946	0.924			
Mean	0.839	0.884	0.943	0.920		0.340	0.272

Spearman correlations were calculated between RSF-habitat ranks and area-adjusted frequencies on a withheld sub-sample of data (20%) 5-times. We also calculated a linear regression between observed frequency and expected RSF scores and assessed the fit. We validated the cattle RSF using a 2-pasture cross validation, where separate cattle RSFs were produced for each pasture and a Spearman rank correlation and linear regression between the two models was calculated in each pasture.

**Figure 3:**
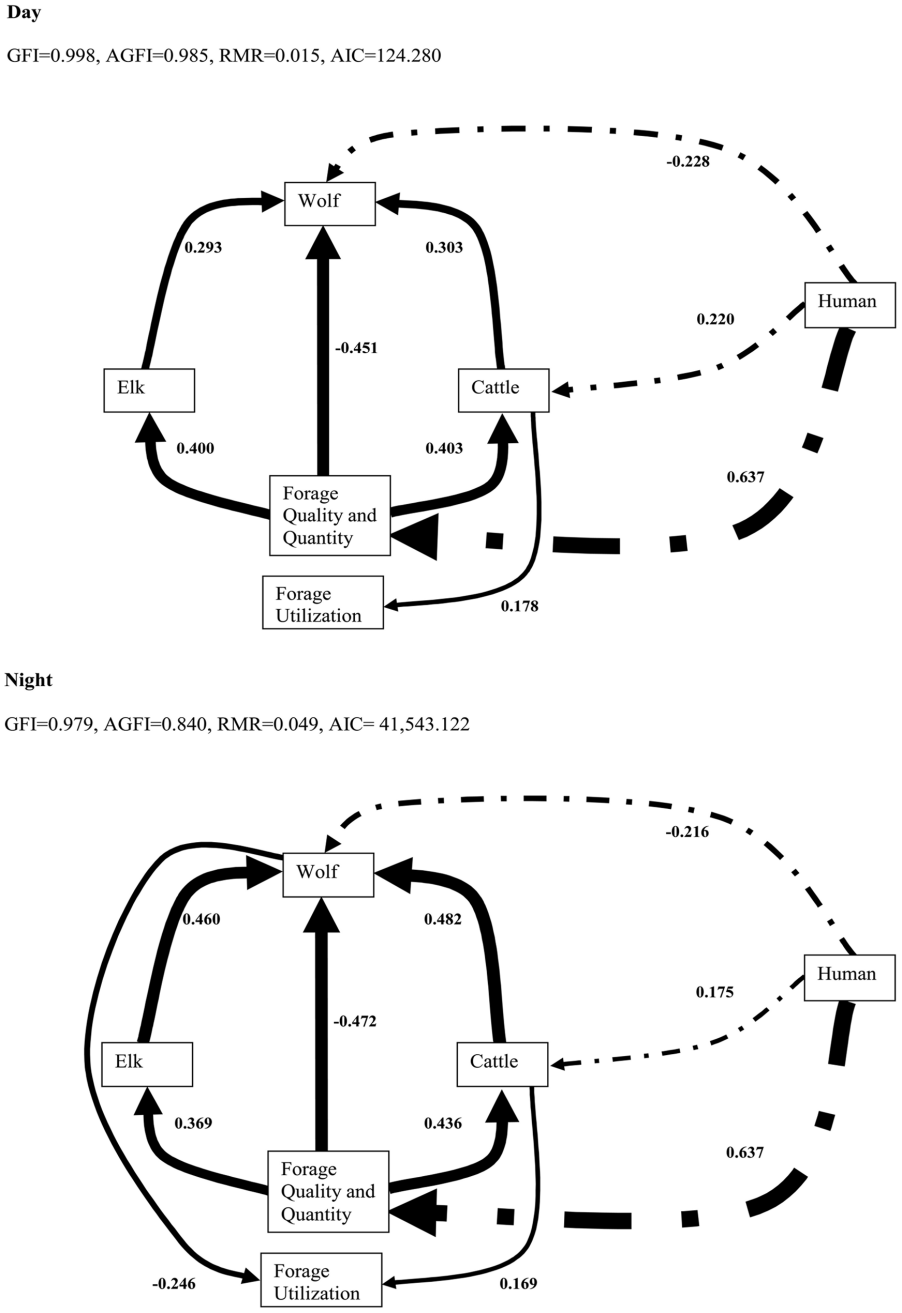
Structural equation model (SEM) illustrating the directions and strengths of relationships among the spatial distribution of humans, wolves, elk, cattle, and forage quality and quantity and forage utilization during the daytime and nighttime in southwest Alberta, Canada. Solid arrows indicate causal direction of the consumer-resource interaction and line thickness is proportional to relationship strength (β coefficient, also indicated). Human influences are represented by dashed-dotted lines.

Our results indicate that the predominant effect of humans was to enhance forage, augmenting herbivores (both native and non-native). In our most parsimonious SEM (Fig. 3), spatial and temporal occurrence of humans was most correlated with occurrence of forage (β = 0.637, *p<*0.0001). Similarly, elk or cattle distribution during the day and the night were correlated with forage (elk day: β = 0.400, *p<*0.0001; elk night: β = 0.369, *p<*0.0001; cattle day: β = 0.403, p<0.0001; cattle night: β = 0.436, *p*<0.0001; Fig. 3). We found positive relationships between the distribution of elk or cattle and wolves during daytime (elk: β = 0.293, *p<*0.0001, cattle: β = 0.303, *p<*0.0001) which strengthened during nighttime when human activity declined (elk: β = 0.460, *p<*0.0001, cattle: β = 0.482, *p<*0.0001; Fig. 3).

In addition to forage, humans had direct effects on the distribution of wolves and cattle (wolves day: β = −0.228, *p<*0.0001; wolves night: β = −0.216, *p<*0.0001; cattle day: β = 0.220, *p*<0.0001; cattle, night: β = 0.175, *p*<0.0001; Fig. 3). Finally, cattle distribution was positively related to forage utilization (β = 0.178, *p<*0.05 during the day; β = 0.169, *p<*0.05 during the night; Fig. 3). We also documented a negative, likely indirect effect of wolves on forage utilization, but only at night (β = −0.246, *p<*0.05; Fig. 3).

**Figure 4:**
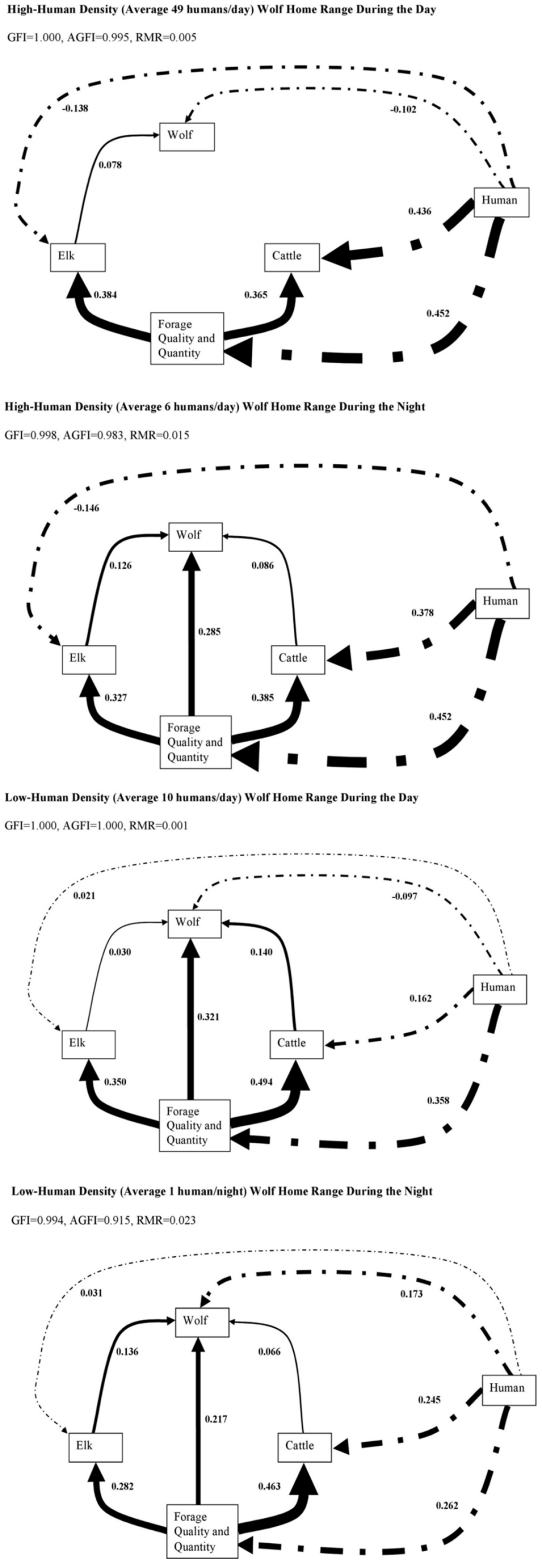
Structural equation model (SEM) illustrating the directions and strengths of relationships among the spatial distribution of humans, wolves, elk, cattle, and forage quality and quantity and forage utilization during the daytime and nighttime within two wolf home ranges with the highest and lowest human density in southwest Alberta, Canada. .

**Figure 5:**
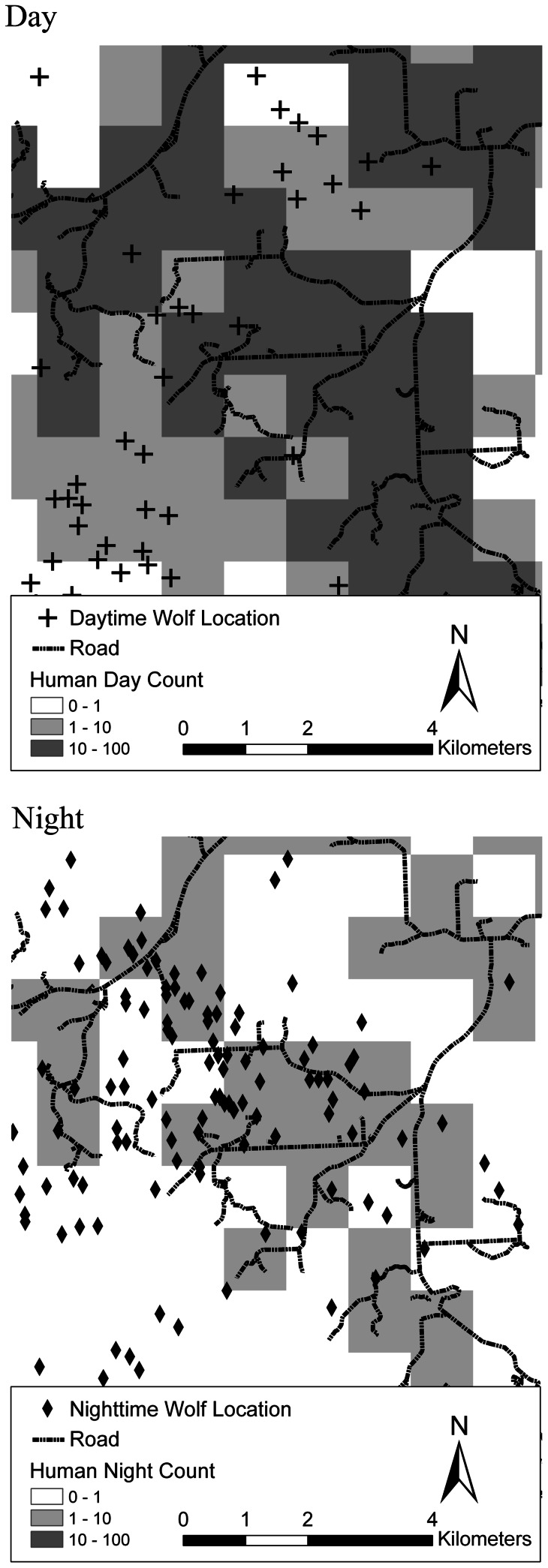
A sample case of wolf telemetry locations around roads with different human counts during the daytime compared to nighttime in southwest Alberta, Canada. Daytime locations are from sunrise to sunset, nighttime locations are sunset to sunrise. Human count is the average number of humans on roads and trails during the day (a), and night (b), calculated from an access index model (Apps et al. 2004). Darker pixels indicate higher human counts.

We found similar patterns to those above within the high and low human density ranges (Fig. 4). Furthermore, human density had a negative effect on wolf distribution during the day (β = −0.102, *p<*0.0001), but not during the night in the high-human density range. Such relationships were also negative during the day (β = −0.097, *p<*0.0001) but positive during the night (β = 0.173, *p<*0.0001) in the low-human density range. This relationship is further illustrated by the difference between day and night locations of wolves monitored in this study. During the night, wolf locations tended to occur near roads (Fig. 5), likely due to low human use over the night.

## Discussion

Here we show the dominant role played by humans in influencing bottom-up vs. top-down dynamics in ecosystems. In particular, our results suggest that human effects on forage could trigger strong bottom-up effects in ecosystems. This contrasts with research conducted in protected areas or relatively intact ecosystems suggesting that human effects on food webs were primarily predator-mediated [1], [2], [4]. However, previous research that addressed interactions between top-down and bottom-up forces found that predator effects may be weak or absent when vegetation productivity is high, at which time bottom-up forces might prevail [31]. Thus, the mechanisms by which humans influenced the food web in our study area may have involved agricultural subsidies that overwhelmed top-down effects. Humans may have positively contributed to forage biomass in our study area through forage crop production and modification of forest to pasture-lands to provide forage for livestock production, an important economic activity. Indeed humans are an important reason why large portions of the world are “green” [32], as agriculture, particularly fertilization, increases nitrogen in the surrounding environment, which positively affects vegetation productivity [33]. In accordance with the mechanisms advocated in such studies, we suggest that human influences on vegetation might prompt bottom-up predominance in ecosystems.

A closer look at the pathways in our SEM also indicated that there were strong, direct influences of humans on all study species at each trophic level. For example, we found evidence of wolf avoidance of people at high levels of human use (a top-down effect). With lower human density there was no effect of humans on wolf distribution, and actually wolves selected for similar habitats as humans. The negative direct effects of humans on wolves was not surprising, because of common lethal control of wolves by humans in the study area in response to livestock predation [34].

The major implication of our results is the need to understand human effects in food webs [35].The predominant human influence on a particular food web may depend on how humans have perturbed the ecosystem [36]. In our study area, forage production was a dominant perturbation. In other areas, other types of human activity may be dominant with different implications for food webs. Understanding human effects in food webs may be particularly important in areas where predator reintroduction has been proposed as a means to restore ecosystems through top-down regulation. For example, predator reintroduction might be ineffective in restoring top-down effects in areas characterized by high densities of humans and livestock because of the predominant bottom-up effect of humans, as shown in our study. Certainly, we do not dispute the importance of top trophic levels in ecosystems, but claims that such effects have primacy worldwide [4] likely underestimate the effects of humans on ecosystems, and in particular may fail to appreciate the importance of resource-mediated effects of humans.

In the past, much emphasis has been placed on direct effects of humans on food webs, for example, through hunting or habitat change. Less emphasis has been placed on measuring indirect effects of humans. For example, conservation-oriented research has typically focused on direct influences of humans on population dynamics of declining and/or rare species. Our study approach emphasizes the importance of considering direct and indirect effects of humans on multi-species interactions and ecosystem dynamics in conservation research. We encourage the application of SEMs as a tool for comparing direct and indirect top-down vs. bottom-up effects of humans on food web dynamics across diverse landscapes. However, we caution that SEMs may allow for testing hypotheses on relationships between species occurrences but do not test the underlying mechanism of those relationships.

The human population is growing worldwide and, to meet its needs, agriculture is not expected to diminish in intensity or distribution. Subsequently, humans will have increasing impacts on ecosystems through a myriad of trophic interactions and pathways. While significant attention has been given to the top-down effects of humans on food webs, careful consideration and quantification of the diversity of direct and indirect effects of humans on multiple species, as we did in this study, will be necessary for effective ecosystem conservation.
